# Sphingosine-1-Phosphate Signaling Regulates Myogenic Responsiveness in Human Resistance Arteries

**DOI:** 10.1371/journal.pone.0138142

**Published:** 2015-09-14

**Authors:** Sonya Hui, Andrew S. Levy, Daniel L. Slack, Marcus J. Burnstein, Lee Errett, Daniel Bonneau, David Latter, Ori D. Rotstein, Steffen-Sebastian Bolz, Darcy Lidington, Julia Voigtlaender-Bolz

**Affiliations:** 1 Toronto Centre for Microvascular Medicine, University of Toronto and St. Michael’s Hospital, Toronto, Canada; 2 Keenan Research Centre for Biomedical Science, St. Michael’s Hospital, Toronto, Canada; 3 Department of Physiology, University of Toronto, Toronto, Canada; 4 Department of Surgery, St. Michael’s Hospital and University of Toronto, Toronto, Canada; 5 Heart & Stroke / Richard Lewar Centre of Excellence in Cardiovascular Research, University of Toronto, Toronto, Canada; 6 Department of Anaesthesia, St. Michael’s Hospital and University of Toronto, Toronto, Canada; Medical University of South Carolina, UNITED STATES

## Abstract

We recently identified sphingosine-1-phosphate (S1P) signaling and the cystic fibrosis transmembrane conductance regulator (CFTR) as prominent regulators of myogenic responsiveness in rodent resistance arteries. However, since rodent models frequently exhibit limitations with respect to human applicability, translation is necessary to validate the relevance of this signaling network for clinical application. We therefore investigated the significance of these regulatory elements in human mesenteric and skeletal muscle resistance arteries. Mesenteric and skeletal muscle resistance arteries were isolated from patient tissue specimens collected during colonic or cardiac bypass surgery. Pressure myography assessments confirmed endothelial integrity, as well as stable phenylephrine and myogenic responses. Both human mesenteric and skeletal muscle resistance arteries (i) express critical S1P signaling elements, (ii) constrict in response to S1P and (iii) lose myogenic responsiveness following S1P receptor antagonism (JTE013). However, while human mesenteric arteries express CFTR, human skeletal muscle resistance arteries do not express detectable levels of CFTR protein. Consequently, modulating CFTR activity enhances myogenic responsiveness only in human mesenteric resistance arteries. We conclude that human mesenteric and skeletal muscle resistance arteries are a reliable and consistent model for translational studies. We demonstrate that the core elements of an S1P-dependent signaling network translate to human mesenteric resistance arteries. Clear species and vascular bed variations are evident, reinforcing the critical need for further translational study.

## Introduction

Rodent models are prevalent research tools; yet their human applicability is strikingly limited [1,2] and translation often lags well-behind basic science advancements. This divide is remarkably pronounced in the resistance artery research field. Despite significant investigative investment, much of our mechanistic understanding of vascular tone control is still limited to rodent models. In particular, the use of isolated human resistance arteries in pressure myography studies, a physiologically relevant means of assessing intact artery function *in vitro*, is disproportionately sparse. This is ostensibly because tissue samples containing relevant vascular beds are difficult, if not impossible, to routinely obtain. However, our dependence on rodent data contributes to the high rate of late-phase clinical trial failures for therapeutics targeting vascular tone and their near-absence in the development pipelines of drug companies.

The present investigation focuses on the myogenic response, a critical mechanism that controls tissue perfusion and peripheral resistance [3–6]. We have recently identified sphingosine-1-phosphate (S1P) signaling and the cystic fibrosis transmembrane conductance regulator (CFTR) as prominent regulators of the myogenic response [7–9]. These elements combine to form a pathophysiologically relevant signaling network, where microvascular CFTR modulates S1P signaling and consequently, underlies clinically relevant functional changes [7,8,10]. These concepts and knowledge have now advanced to a stage where translation to the human setting is mandatory to validate and ultimately exploit this mechanism’s therapeutic potential. In fact, a recent editorial highlighting this work has legitimately challenged the inherent premise that S1P signaling is an equally prominent regulator of myogenic tone within the human microcirculation [11].

In this investigation, we assess whether S1P signaling modulates human resistance artery myogenic tone. Since myogenic mechanisms display significant heterogeneity across vascular beds [12], our assessment spans 2 distinct artery types: mesenteric and skeletal muscle. As rationale for these selections, mouse mesenteric arteries are widely used experimentally, while skeletal muscle resistance arteries possess significant physiological relevance, since they are prominent regulators of blood pressure and blood distribution [13–15]. As our core hypothesis, we propose that the S1P/CFTR signaling axis [7] prominently regulates myogenic tone in both human mesenteric and human skeletal muscle resistance arteries.

## Materials and Methods

The use of human subjects in this study conforms to the principles outlined in the *Declaration of Helsinki* and was approved by the Research Ethics Board of St. Michael’s Hospital, Toronto, Canada (Approval #11–198). Patients 18 years of age and older who planned to undergo elective surgery were recruited from the outpatient general surgery (mesenteric resistance arteries) or cardiac surgery (skeletal muscle resistance arteries) clinics at St. Michael’s Hospital. All patients provided informed written consent through Research Ethics Board-approved consent forms prior to study enrolment.

The use of animals in this investigation conforms to the *Guide for the Care and Use of Laboratory Animals* published by the NIH (Publication No. 85–23, revised 1996); the experimental protocols were approved by the Institutional Animal Care and Use Committees at the University of Toronto and were conducted in accordance with Canadian animal protection laws.

### Isolation of human resistance arteries

A total of 37 general surgery patients receiving bowel resection surgery were included: 27 were treated for colon cancer; the remaining cases involved other pathologies, including Crohn’s disease, ulcerative colitis, diverticular disease and fistulas. A total of 51 cardiac surgery patients receiving coronary artery bypass graft surgery were included: all patients were diagnosed with coronary artery disease and most patients had an ejection fraction greater than 40%. A complete composite of patient characteristics, co-morbidities and treatments is provided in Tables A and B in S1 File.

Surgeons directly provided a small piece of either human mesentery (4–5cm^3^) or thoracic wall skeletal muscle (3–4cm^3^) to research staff inside the operating room. The surgeon identified and excised a small section of tissue without cautery (the use of cautery damages arteries within the sample). The specimen was immersed in room temperature MOPS buffer, placed on ice and transported to the laboratory; once the specimen cooled, it was washed with ice-cold MOPS buffer and placed in a Petri dish. Resistance arteries were carefully dissected from the surrounding tissue, taking care to minimize vessel tension during the isolation process. Vessel segments (~1mm in length) were collected and either (i) functionally assessed by pressure myography [8] or (ii) used in standard western blotting / qRT-PCR procedures [7].

### Functional assessment

Resistance arteries were cannulated, pressurized to 45mmHg and slowly warmed to 37°C (over 30 minutes). The transmural pressure (TMP) was increased to 60mmHg; following a stabilization period of 30 minutes, vessel viability was assessed with either a single-dose of 10μmol/L phenylephrine (PE) or 60μmol/L KCl. Vessels that failed to constrict at least 30% were considered compromised and excluded.

Phenylephrine- and S1P-stimulated vasomotor responses were quantified as tone = [(dia_max_−dia_response_)/dia_max_]×100, where dia_response_ refers to the steady-state diameter following agent application and dia_max_ refers to the maximal vessel diameter (measured under calcium-free conditions). All vasomotor responses were assessed at a transmural pressure of 60 mmHg.

Myogenic responsiveness was assessed as either: (i) the steady-state level of constriction relative to the passive diameter over a range of 20–100mmHg in 20mmHg increments (defined here as “*myogenic tone*”) [7,8,10] or (ii) magnitude of constriction that follows a large, single pressure step of 60mmHg to 100mmHg (defined here as “*active myogenic constriction*”) [9,16,17]. For the former, myogenic tone was calculated as tone = [(dia_max_−dia_active_)/dia_max_]×100, where dia_active_ refers to the minimal diameter observed within 8-minutes post-pressure step and dia_max_ refers to the maximal vessel diameter (measured under calcium-free conditions). For the latter, active myogenic constriction was calculated as dia_initial_-dia_min_, where dia_initial_ is the diameter immediately following the pressure step and dia_min_ is the minimal diameter observed within 8 minutes post-pressure step.

### Isolation of mouse resistance arteries

Wild-type male mice (2–3 months; C57BL/6N) were purchased from Charles River Laboratories (Montreal, Canada). Prior to experiments, mice were housed for two weeks under a standard 14h:10h light-dark cycle, fed normal chow and had access to water *ad libitum*. Following full anesthesia with isoflurane, the animal was euthanized by cervical dislocation. The mesentery and cremaster muscles were grossly dissected and placed in a Petri dish containing ice-cold MOPS buffer. Mesenteric arteries and cremaster skeletal muscle resistance arteries were carefully dissected and immediately snap-frozen with liquid nitrogen (for PCR assessments) [8].

### Molecular and Biochemical Procedures

Standard procedures were used for quantitative RT-PCR and western blots [7]. Specific methodological details are provided in the S1 File. The Diagnostic Laboratories at St. Michael’s Hospital quantified serum “NT-proBNP” levels, using an electrochemiluminescence assay (Elecsys proBNP II assay automated on a cobas e601 platform; Roche Diagnostics; Indianapolis, USA). “NT-proBNP” is a N-terminal cleavage peptide that is generated when the prohormone form of B-type natriuretic peptide (BNP) is converted into an active hormone: it is a biomarker of compromised cardiac function that is used to screen/diagnose acute heart failure [18]. As per clinical guidelines utilized at St. Michael’s Hospital, patients in ambulatory care were considered to have a positive result (i.e., heart failure; “BNP+”) if serum NT-proBNP values were: (i) above 125ng/L in patients ≤50 years, (ii) above 250ng/L in patients 50–75 years or (iii) above 500ng/L in patients over 75 years.

### Statistics

All data are expressed as means ± SEM; unless otherwise specified, n is the number of vessels studied and N is the number of patients. Statistical comparisons utilized the following procedures: a non-parametric (i.e., exact p-value computation) Mann-Whitney test for one unpaired comparison; a non-parametric Wilcoxon test for one paired comparison; a nonparametric Friedman repeated measures one-way ANOVA for multiple paired comparisons, in conjunction with a Dunn’s multiple comparison correction; and a two-way ANOVA for comparing myogenic tone and phenylephrine dose-response relationships (i.e., differences between curves). Differences were considered significant at P<0.05.

## Results

### Quality control

Data derived from human mesenteric and skeletal muscle resistance arteries are sparse: it was, therefore, crucial to rigorously assess both vessel quality (i.e., endothelial and smooth muscle function) and response stability. A large proportion of both human mesenteric and skeletal muscle resistance arteries failed initial viability tests: our overall success rate was approximately 17% for both artery types.

**Fig 1:**
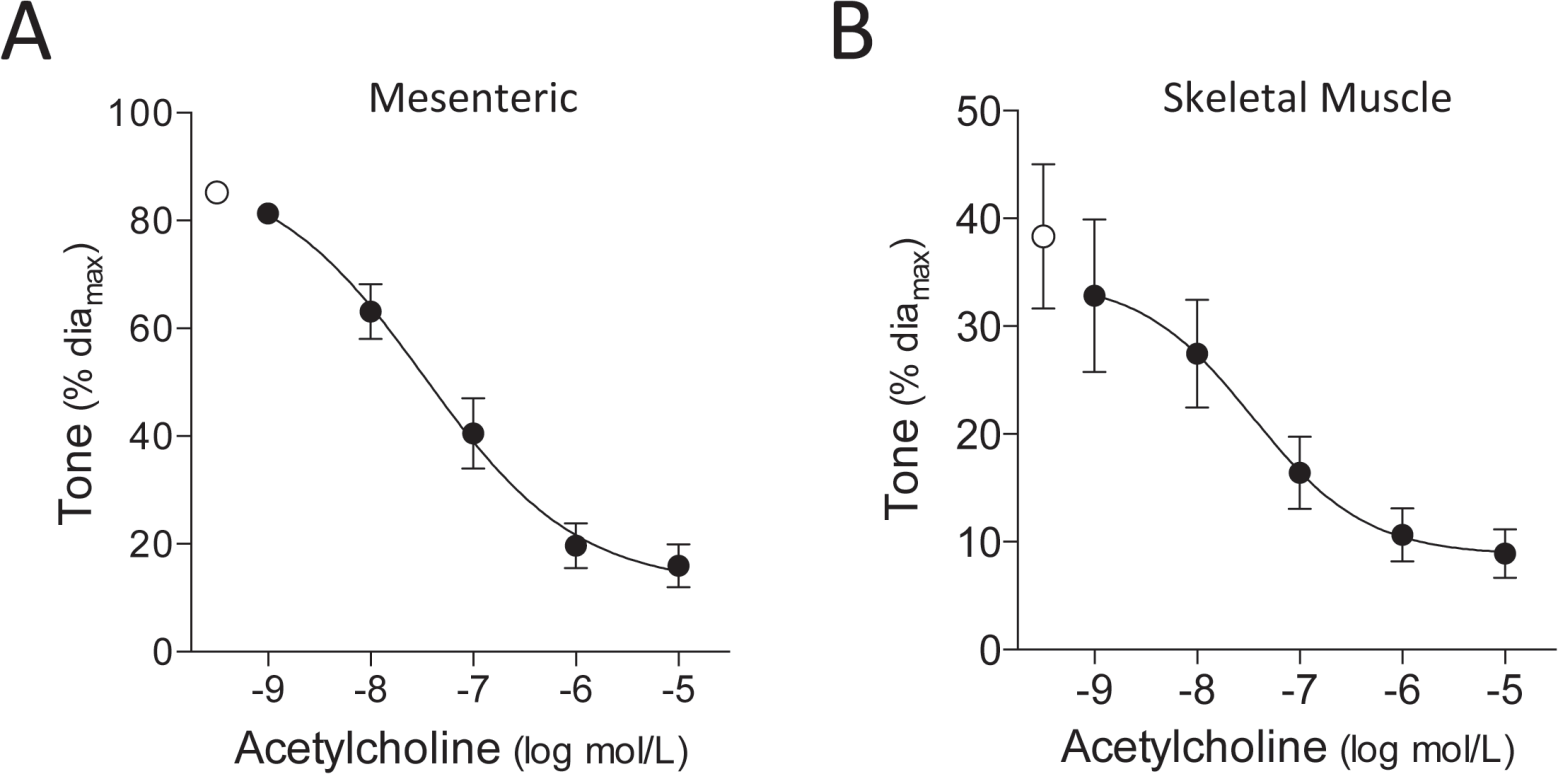
Human mesenteric and skeletal muscle resistance artery vasodilator responses. Human (A) mesenteric (n = 7 vessels from N = 3 patients) and (B) skeletal muscle (n = 8, N = 5) resistance arteries constrict in response to 10^-5^ mol/L phenylephrine (open circles) and dose-dependently dilate when acetylcholine is subsequently co-applied (closed circles). For mesenteric arteries: dia_max_ = 255±23μm and logEC_50_ = -7.4±0.2. For skeletal muscle arteries: dia_max_ = 133±22μm and logEC_50_ = -7.2±0.2. Dia_max_ is defined as the maximal diameter (under calcium free buffer conditions) at 60mmHg.

We confirmed smooth muscle and endothelial functional integrity in both human mesenteric and skeletal muscle resistance arteries, by demonstrating constriction in response to 10μmol/L phenylephrine (PE) and dose-dependent vasodilatation to subsequent acetylcholine (Ach) co-application (Fig 1). Since many tissue samples are not available until late in the day, we determined whether overnight storage at 4°C (in MOPS buffer) influences artery responses (which would allow us to shift lengthy experimental protocols to within normal working hours). Human mesenteric artery responses are unaffected (Fig A in S1 File) and therefore, we incorporated overnight storage as a standard procedure for all samples. In contrast, human skeletal muscle artery responses changed following storage (Fig A in S1 File) and therefore, experiments proceeded immediately following vessel isolation.

**Fig 2:**
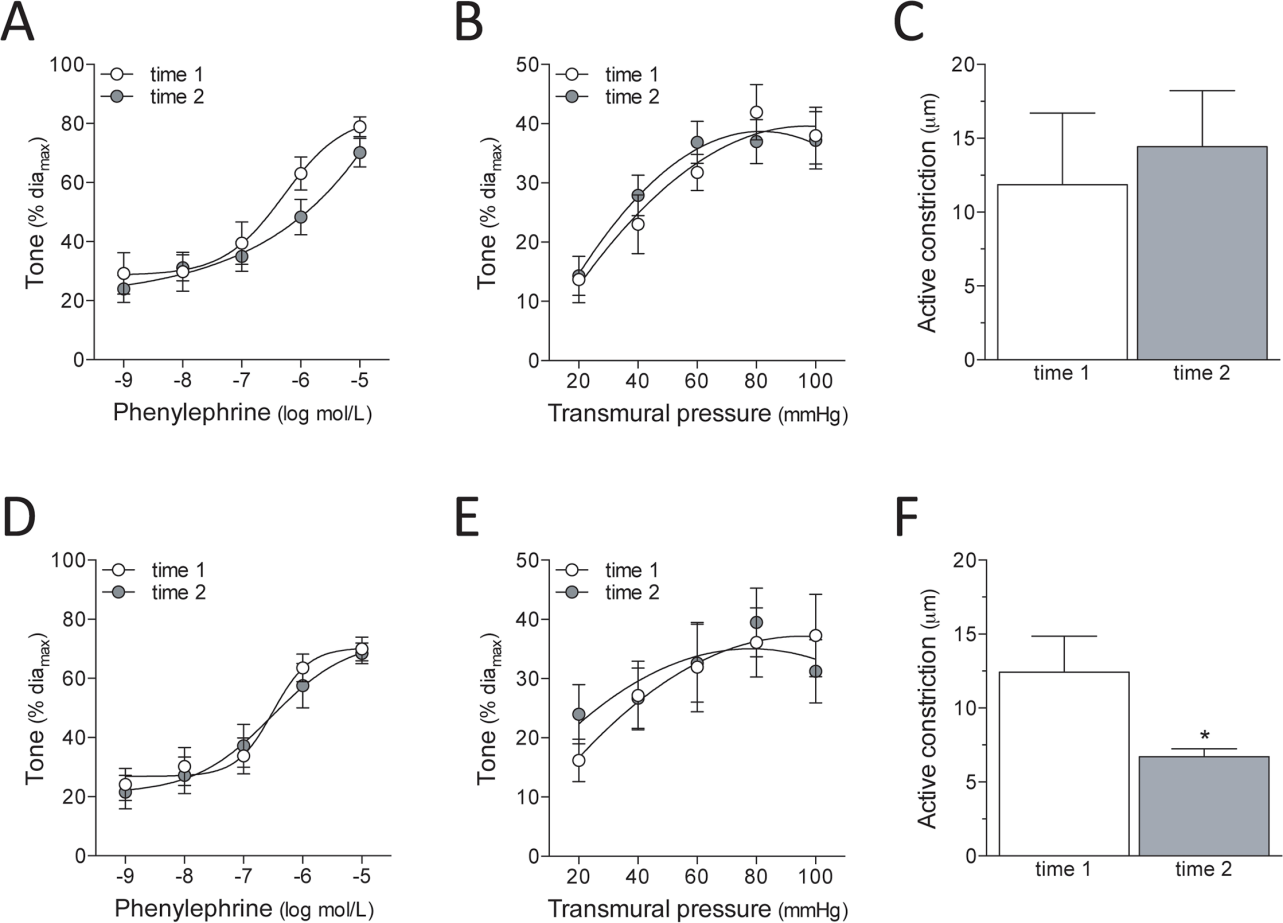
Response stability in human mesenteric and skeletal muscle resistance arteries. Time-control experiments confirm that (A) phenylephrine (PE)-stimulated vasoconstriction (logEC_50_ = -6.4±0.2; dia_max_ = 173±33μm; n = 7 vessels from N = 5 patients), (B) myogenic tone (dia_max_ = 185±34μm; n = 7, N = 4) and (C) active myogenic constriction (following a pressure step from 60mmHg to 100mmHg; dia_max_ = 105±15μm; n = 7, N = 3) are consistent in human mesenteric resistance arteries over two separate assessments. (D) PE-stimulated vasoconstriction (logEC_50_ = -6.5±0.1; dia_max_ = 104±13μm; n = 10, N = 10) and (E) myogenic tone (dia_max_ = 122±21μm; n = 9, N = 8) are also consistent over two separate assessments in human skeletal muscle resistance arteries; however, (F) active myogenic constriction (dia_max_ = 94±9μm; n = 7, N = 6) is not stable. Dia_max_ is defined as the maximal diameter (under calcium free buffer conditions) at 60mmHg. Myogenic tone (Panels A and D) and PE responses (Panels B and E) were statistically compared with a paired two-way ANOVA; active constriction measures (Panels C and F) were compared with a Wilcoxon test. * denotes P<0.05 for a paired comparisons.

Time-control experiments determined whether PE and myogenic responses were sufficiently stable for paired comparison experimental designs (i.e., pre/post inhibitor treatment): functional parameters were initially assessed (“time 1”); the vessel was then incubated for 30 minutes under normal buffer conditions (i.e., the time period that corresponds to a typical inhibitor incubation requirement) and re-tested (“time 2”). Human mesenteric resistance artery responses are stable (Fig 2); indeed, they remain comparable after a second 30-minute incubation period and re-test (Fig B in S1 File). Since measuring active constriction requires substantially less procedural time than for myogenic tone, we utilized the former in mesenteric artery inhibitor studies.

Human skeletal muscle resistance artery responses are less stable, relative to human mesenteric arteries: although PE responses and myogenic tone are unchanged at “time 2”, active myogenic vasoconstriction is significantly reduced (Fig 2); myogenic tone is not stable beyond “time 2” (Fig B in S1 File). Inhibitor studies using skeletal arteries, therefore, required the myogenic tone assessment procedure.

We confirmed that neither the type of surgical procedure (open versus laparoscopic for mesenteric arteries; Fig C in S1 File), nor the operating surgeon (Fig D in S1 File) influenced functional responses. The maximal artery diameter range was approximately 200–250μm (mesenteric artery dia_max_ range = 59μm-318μm; skeletal muscle artery dia_max_ range = 33μm-217μm); responses were similar in the upper and lower quartiles of maximal vessel diameter (Fig E in S1 File).

### Sphingosine-1-phosphate signaling in human mesenteric resistance arteries

**Fig 3:**
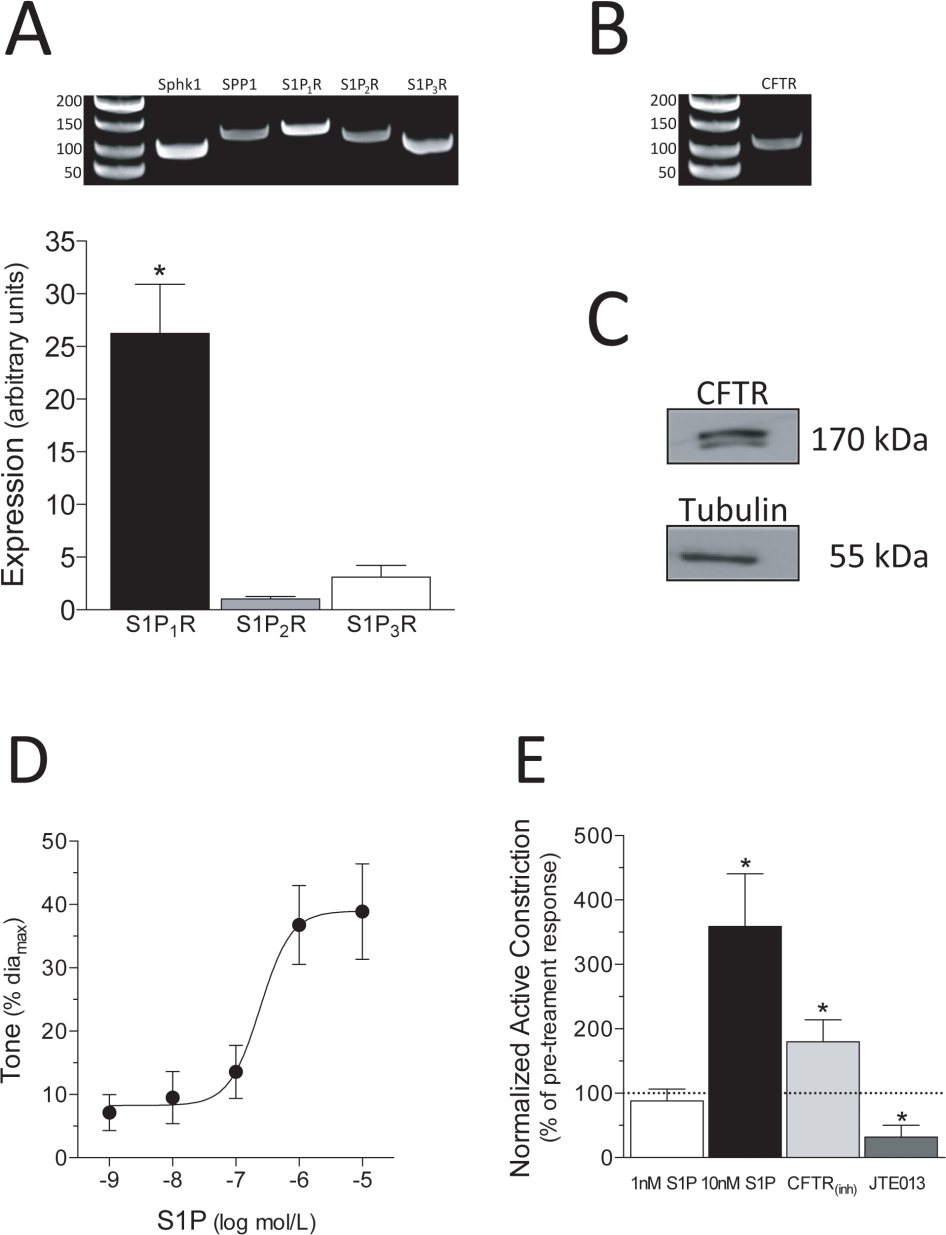
S1P signaling in human mesenteric resistance arteries. (A) Human mesenteric resistance arteries express mRNA encoding critical sphingosine-1-phosphate (S1P) signaling elements (N = 8 patients), including sphingosine kinase 1 (Sphk1), S1P phosphohydrolase 1 (SPP1) and S1P receptor subtypes 1–3 (S1P_1_R, S1P_2_R and S1P_3_R). In terms of relative expression, S1P_1_R is the most highly-expressed receptor, followed by S1P_3_R and then S1P_2_R (N = 8). Human mesenteric arteries express the cystic fibrosis transmembrane conductance regulator (CFTR) at both the (B) mRNA (N = 8) and (C) protein levels (N = 3); in (C), tubulin confirms adequate loading. (D) S1P stimulates dose-dependent vasoconstriction (logEC_50_ = -6.7±0.2; dia_max_ = 121±13μm; n = 5 vessels from N = 3 patients). (E) Shown are normalized active constriction measurements (post-treatment measures normalized to pre-treatment responses). At sub-threshold concentrations (i.e., levels that do not stimulate overt constriction), S1P dose-dependently enhances myogenic vasoconstriction (1nmol/L S1P: dia_max_ = 177±27μm, n = 7, N = 5; 10nmol/L S1P: dia_max_ = 165±19μm, n = 11, N = 6); CFTR inhibition (1nmol/L CFTR_(inh)_-172: dia_max_ = 203±13μm, n = 12, N = 6) also enhances myogenic vasoconstriction, while S1P receptor antagonism (10nmol/L JTE013: dia_max_ = 148±30μm, n = 7, N = 4) attenuates myogenic vasoconstriction. Dia_max_ is defined as the maximal diameter (under calcium free buffer conditions) at 60mmHg. In Panel A, * denotes P<0.05 for S1P_1_R relative to the other two receptors (Friedman ANOVA); in Panel E, * denotes P<0.05 for a paired comparison to the pre-treatment response (Wilcoxon test).

Using end-point and quantitative PCR (qPCR), we demonstrate that human mesenteric resistance arteries express mRNA encoding all critical S1P signaling components, including sphingosine kinase 1 (Sphk1), S1P phosphohydrolase 1 (SPP1) and S1P receptors (S1PR) subtypes 1–3 (Fig 3A; N = 8 separate patient samples). The relative S1P receptor mRNA expression pattern in human mesenteric arteries (S1P_1_R >> S1P_3_R = S1P_2_R; Fig 3A) is similar to that of mouse mesenteric arteries (Panel A of Fig F in S1 File), with the exception that S1P_1_R is more highly expressed relative to S1P_2_R in human arteries (25 fold higher in human arteries versus 10 fold higher in mouse arteries). Human mesenteric resistance arteries express CFTR at both the mRNA (Fig 3B; representative of N = 8) and protein (Fig 3C; representative of N = 3) levels. Consistent with data reported in mice [7,8,10], S1P stimulates dose-dependent vasoconstriction in human mesenteric arteries (Fig 3D) and dose-dependently enhances myogenic vasoconstriction (Fig 3E); inhibiting CFTR channel activity (1nmol/L CFTR_(inh)_-172) also augments myogenic vasoconstriction. We antagonized S1P signaling in human arteries with the chemical antagonist JTE013; we selected this inhibitor based on its extensive use in our previous investigations in rodents [8–10,16,19–21]. JTE013 is a purported S1P_2_R-selective antagonist; however, as scrutinized in our discussion (see below), emerging evidence suggests that its receptor subtype selectivity is imperfect [10,22,23]. Thus, we use JTE013 to assess S1P signaling in human arteries, without specifically attributing the effects to a particular S1P receptor subtype. Consistent with previous observations in rodents [8], JTE013 (10nmol/L) attenuates myogenic vasoconstriction in human mesenteric arteries (Fig 3E), without causing a general loss in contractility (PE responses are unaffected; Fig G in S1 File).

### Sphingosine-1-phosphate signaling in human skeletal muscle resistance arteries

Although all of the human skeletal muscle tissue specimens originated from patients requiring cardiac surgery, our patient cohort varied in terms of their cardiac function. Since experimental heart failure modifies skeletal muscle artery myogenic tone [8], we first determined whether the heterogeneity in cardiac function within our patient cohort influenced myogenic tone. As shown in Fig H of the S1 File, human skeletal muscle resistance artery myogenic tone did not differ when segregated by (i) serum NT-proBNP levels (positive versus negative result) [18]; (ii) New York Heart Association (NYHA) functional class (Class I versus Classes 2 and above) [24,25]; (iii) the presence of reduced left ventricular ejection fraction (normal defined as ≥55%) [25]; or (iv) previous history of myocardial infarction. The consistency in myogenic responsiveness confirms that our model of human skeletal muscle resistance arteries is highly suited for mechanistic inspection.

**Fig 4:**
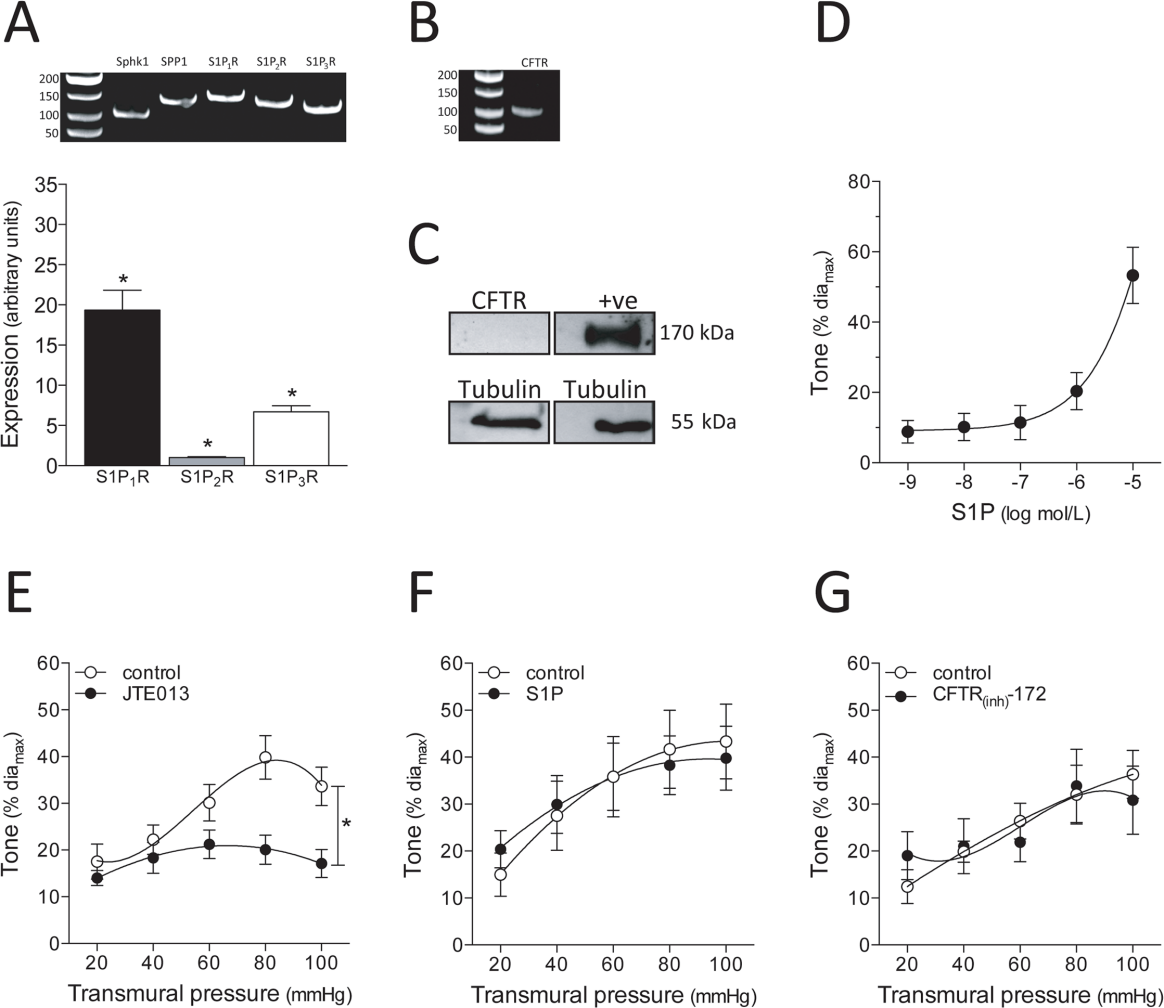
S1P signaling in human skeletal muscle resistance arteries. (A) Human skeletal muscle resistance arteries express mRNA encoding critical sphingosine-1-phosphate (S1P) signaling elements (N = 8 patient samples), including sphingosine kinase 1 (Sphk1), S1P phosphohydrolase 1 (SPP1) and S1P receptor subtypes 1–3 (S1P_1_R, S1P_2_R and S1P_3_R). In terms of relative expression, S1P_1_R is the most highly expressed; S1P_3_R is more highly expressed than S1P_2_R (N = 8). Human skeletal muscle resistance arteries (B) express detectable levels of cystic fibrosis transmembrane conductance regulator (CFTR) mRNA (N = 8); however, (C) CFTR protein is not detected (N = 5). In (C), tubulin confirms adequate loading. (D) S1P stimulates dose-dependent vasoconstriction (dia_max_ = 122.2±16.5μm; n = 6 vessels from N = 4 patients). (E) S1P receptor antagonism (100pmol/L JTE013: dia_max_ = 132±15μm, n = 8, N = 5) attenuates myogenic tone. However, neither (F) sub-threshold concentrations of S1P (10nmol/L S1P: dia_max_ = 110±20μm; n = 5, N = 4) nor (G) CFTR inhibition (10pmol/L CFTR_(inh)_-172: dia_max_ = 139±33μm, n = 5, N = 4) modulate myogenic tone. Dia_max_ is defined as the maximal diameter (under calcium free buffer conditions) at 60mmHg. In Panel A, * denotes P<0.05 relative to all other receptor subtypes (Friedman ANOVA). Panels E, F and G were statistically compared with a paired two-way ANOVA; in Panel E, * denotes P<0.05 relative to the pre-treatment control response.

As in human mesenteric arteries, human skeletal muscle resistance arteries express mRNA encoding Sphk1, SPP1 and S1P receptors 1–3 (Fig 4A, N = 8 separate patient samples). Notably, the S1P receptor mRNA expression pattern (S1P_1_R > S1P_3_R > S1P_2_R) does not match that of human mesenteric arteries (compare Fig 3A and Fig 4A). As observed for mesenteric arteries, S1P_1_R expression is significantly higher in human skeletal muscle arteries, relative to mouse cremaster skeletal muscle arteries (Panel B of Fig F in S1 File). Human skeletal muscle resistance arteries express mRNA encoding CFTR (Fig 4B, representative of N = 7); however, the expression level is 34 fold lower than in mesenteric arteries (P<0.05; Mann-Whitney test). CFTR protein was not detected in human skeletal muscle arteries (Fig 4C, representative of N = 5). As in human mesenteric arteries, (i) human skeletal muscle resistance arteries dose-dependently constrict in response to S1P (Fig 4D) and (ii) antagonizing S1P receptors (0.1nmol/L JTE013) attenuates myogenic responsiveness (Fig 4E), without affecting PE responses (Fig I in S1 File). However, in contrast to human mesenteric arteries (Fig 3), neither 10nmol/L S1P (Fig 4F) nor CFTR inhibition (10pmol/L CFTR_(inh)_-172; Fig 4G) specifically enhanced myogenic responses. In the case of the latter, increasing the CFTR inhibitor concentration beyond 10pmol/L attenuated both PE and myogenic responses (Fig J in S1 File).

## Discussion

We have previously demonstrated that CFTR and S1P signaling are critical signaling elements that regulate rodent resistance artery function in health and experimental disease models [7–10]. As a prerequisite for capitalizing on this knowledge for human gain, we established standardized procedures for assessing human mesenteric and skeletal muscle resistance arteries, in order to translate the key aspects of the signaling mechanism.

Even with strict procedures for obtaining and handling the tissue samples, 75% of the arteries tested failed our viability standards. However, these rigorous constraints ensured the inclusion of high quality specimens, as evidenced by robust, reproducible and stable vasomotor and myogenic responses. In fact, reliably demonstrating human mesenteric artery PE and Ach responses is, in relation to present literature [26], a significant achievement. Despite the inherent variability within the recruited patient population, resistance artery myogenic responses were reasonably consistent: this unexpected characteristic permits manageable sample sizes.

The myogenic response serves several critical roles in the microcirculation, including: preserving constant tissue perfusion at varying systemic pressures (autoregulation) [3,4], maintaining proper capillary hydrostatic pressure [27,28] and, by generating a significant proportion of peripheral resistance, contributing to systemic blood pressure control [5,6]. The complex molecular mechanisms that underpin this response display remarkable heterogeneity across species and vascular bed (reviewed by Lidington et al. [12]): this obstacle has undoubtedly hindered the development of pharmaceuticals that specifically target microvascular tone (e.g., as an anti-hypertensive agent). A significant strength of the current investigation, therefore, is that it documents the mechanistic differences in human arteries derived from two distinct vascular beds.

Our molecular, biochemical and functional data clearly demonstrate that S1P signaling is a dominant regulator of myogenic vasoconstriction in both human mesenteric and skeletal muscle resistance arteries. Specifically, we show that: (i) the critical signaling elements are present; (ii) S1P stimulates vasoconstriction and (iii) S1P receptor antagonism (JTE013) attenuates myogenic responsiveness. As a cornerstone of myogenic vasoconstriction, S1P signaling represents a potential therapeutic target in at least two human microvascular beds; our rodent data [8,10,20,21] predict that this applicability likely extends beyond just two microvascular beds.

Although JTE013 is a purported S1P_2_ receptor-selective antagonist, emerging evidence indicates that it is also capable of antagonizing other S1P receptor subtypes [10,22,23]. There is no indication that JTE013 has effects beyond S1P signaling: in our hands, other agonist responses, including endothelin-1 and phenylephrine, are fully preserved [8,10,21]. Thus, as we have in our previous studies [10,21], we reconcile JTE013’s specificity issue by asserting that it is suitable for investigating S1P signaling, while strongly urging caution in terms of attributing its effects solely to S1P_2_R. From a translational perspective, our study demonstrates that myogenic vasoconstriction in human mesenteric and skeletal muscle resistance arteries is (i) sensitive to JTE013-mediated antagonism and therefore, (ii) dependent on S1P signaling: these results and conclusions are entirely consistent with those made in rodent models [8–10,16,19–21].

Unlike the S1P signaling components, microvascular CFTR is differentially expressed in the two artery types; consequently, CFTR modulates myogenic vasoconstriction only in human mesenteric arteries. The lack of CFTR protein expression in human skeletal muscle resistance arteries is surprising, given that CFTR was first characterized as a myogenic modulator in hamster skeletal muscle resistance arteries [9]. Species heterogeneity is one plausible explanation for the discrepancy. However, we have previously shown that experimental heart failure down-regulates CFTR expression in mice [7]: we therefore cannot exclude that the patient’s medical condition significantly contributed to the observed phenotype.

Microvascular CFTR expression may be necessary for S1P to modulate resistance artery myogenic tone. We recently demonstrated that exogenous S1P inhibits CFTR channel activity by an S1P_1_ receptor-dependent mechanism [29]. Since the S1P_1_ receptor is the most highly expressed subtype, we assert that S1P attenuates CFTR activity at S1P levels that do not stimulate overt vasoconstriction. Importantly, our proposed model dictates that the sensitivity to S1P requires CFTR expression: hence, S1P augments myogenic tone in human mesenteric arteries, but not in human skeletal muscle resistance arteries. Accordingly, direct CFTR (CFTR_(inh)_-172) inhibition mirrors the effects of S1P: it augments myogenic responses in mesenteric arteries, but has no effect in skeletal muscle resistance arteries.

There is also a striking disparity between rodent and human resistance arteries in terms of inhibitor sensitivity: both JTE013 and CFTR(inh)-172 are **at least** 100-fold more potent in human arteries. In fact, at the concentrations utilized in our rodent studies [7–9,16], both inhibitors non-selectively abolished contractility (data not shown). Even S1P exhibits at least 100-fold higher potency in human mesenteric arteries, relative to its murine counterpart [8].

Thus, while our investigation identifies S1P signaling as a fundamental myogenic regulator in humans, it also defines several significant **dissimilarities** that directly bear on our mechanistic understanding, dosage selection and ultimately, the overall development of new therapeutic strategies. Species and vascular bed differences may explain some of the discrepancies; however, the undeniable issue with patient samples is discerning the differences caused by underlying medical conditions. This revelation reinforces the critical need for translation and yet sets limits on the implications of any individual study. We demonstrate that high quality microvascular assessments at the patient level are achievable; yet the success rate (25–30%) and ancillary requirements (e.g., recruiting patients) demand considerable investigative investment in return for these human data. Thus, an expansive translational program clearly hinges on developing new technologies that integrate automation [30] and improve experimental throughput.

In summary, we demonstrate that human mesenteric and skeletal muscle resistance arteries are, in principle, a reliable and consistent model for translating myogenic mechanisms. Accordingly, we demonstrate that S1P signaling is a core modulator of myogenic responsiveness in human resistance arteries, as initially identified in rodent models [7–10]. Although clear species and vascular bed differences are evident, we substantiate the speculated value of pharmacologically targeting S1P signaling within the microvascular system. Since S1P-modulating therapeutics are either in clinical use (Fingolimod) or development, our seminal study provides justification for advancing these medications as efficacious human cardiovascular system modulators.

## Supporting Information

S1 File Supplemental Methods and Results. This complete supplemental information file contains: (i) reagent information; (ii) methodological details pertaining to RNA isolation, quantitative PCR and western blotting; (iii) supplemental tables documenting patient characteristics (Tables A and B) and PCR primers (Tables C and D); and (iv) supplemental data (Figs A-J).
(PDF)
