# Supplementary material for: Sphingosine-1-Phosphate Signaling Regulates Myogenic Responsiveness in Human Resistance Arteries
Source: PLoS One. 2015 Sep 14;10(9):e0138142. doi: 10.1371/journal.pone.0138142 (PMC4569583; doi:10.1371/journal.pone.0138142)
Supplement: S1 File — This complete supplemental information file contains: (i) reagent information; (ii) methodological details pertaining to RNA isolation, quantitative PCR and western blotting; (iii) supplemental tables documenting patient characteristics (Tables A and B) and PCR primers (Tables C and D); and (iv) supplemental data (Figs A-J). (PDF) [file pone.0138142.s001.pdf]

Supplemental Methods and Results

Reagents

Unless specified, reagents were purchased from *Bioshop Canada* (Burlington, Canada). $CFTR_{(inh)}$-172, acetylcholine (Ach) and phenylephrine (PE) were purchased from Sigma-Aldrich (Oakville, Canada); sphingosine-1-phosphate (S1P) was purchased from *Biomol International* (distributed by *Cedarlane Laboratories*; Burlington, Canada); and JTE013 was purchased from Tocris Bioscience (*Cedarlane Laboratories*). The MOPS-buffered saline solution (MOPS buffer) contained [mmol/L]: NaCl 145, KCl 4.7, $CaCl_2$ 3.0, $MgSO_4$ •$7H_2O$ 1.17, NaH_2PO_4 •$2H_2O$ 1.2, pyruvate 2.0, EDTA 0.02, MOPS (3-morpholinopropanesulfonic acid) 3.0, and glucose 5.0.

RNA Isolation and Reverse Transcription

Resistance artery RNA was isolated with *Norgen Biotek* (Thorold, Canada) "Total RNA Purification Micro" spin columns, using the proteinase K digestion and DNA removal procedures, as directed by the manufacturer's instructions. The eluted RNA was quantified with an *Agilent Technologies* RNA 6000 Pico Kit and Bioanalyzer; the analysis confirmed that high-quality RNA was retrieved from all samples. RNA was converted to cDNA using a "Superscript III" reverse transcription kit (*Invitrogen Life Technologies*; Burlington, Canada), according to the manufacturer's directions. Residual RNA was removed by incubating the cDNA with RNAse H (0.125U/µl; *New England Biolabs Canada*; Whitby, Canada).

Quantitative and End-point PCR

Quantitative PCR was performed using an *Applied Biosystems* Viia7 Real Time PCR system and Power SYBR® Green PCR master mix (both distributed by *Invitrogen Life Technologies*). Each primer set (400nmol/L in each reaction; Tables C and D) was rigorously validated to ensure specificity and comparable efficiency. Gene targets were assessed in triplicate, using 1ng of cDNA generated from the reverse transcription; negative controls received water. The PCR amplification consisted of 10 minutes denaturation at 95°C, followed by 40 cycles of amplification (15s at 95°C + 60s at 60°C). Following amplification, the amplicons were melted: the resulting dissociation curve confirmed that only a single product was produced.

S1P receptor transcript expression levels were calculated from the ΔCt values relative to the standard housekeeping gene hydroxymethylbilane synthase (HMBS). To confirm that HMBS was reliable for normalization, transcript expression levels were also calculated from ΔCt values relative to glyceraldehyde 3-phosphate dehydrogenase (GAPDH) and glucose-6-phosphate dehydrogenase (G6PD): similar results were returned in both cases.

End-point PCR reactions were performed using an i-Taq™ DNA polymerase kit (*FroggaBio*, Toronto, Canada), according to manufacturer's instructions. The primer sequences (Tables C and D), primer concentration (400nmol/L) and cDNA load (1ng) were the same as for quantitative PCR reactions. The PCR amplification protocol consisted of 5 minutes denaturation at 95°C, followed by 35 cycles of amplification (60s at 95°C + 30s at 60°C + 30s at 72°C). The PCR products were electrophoresed on a 10% acrylamide DNA gel, stained with SYBR Safe DNA Gel Stain (*Invitrogen Life Technologies*) and imaged by UV transillumination.

Western Blotting

Western blots for CFTR were completed as previously described [1]. Briefly, lysates were prepared by grinding resistance arteries in lysis buffer containing 25mmol/L Tris (pH6.8), 1% SDS, 10% glycerol, 1mmol/L EDTA, 0.7mol/L β-mercaptoethanol and 25μg/ml protease inhibitor cocktail. The resulting homogenates were subjected to 5 freeze-thaw cycles using liquid nitrogen, heated to 65°C for 10 minutes and then centrifuged (30 minutes at 15,000g; 4°C). Proteins were separated electrophoretically on 5% acrylamide gels and transferred onto polyvinylidene difluoride (PVDF) membranes.

The membranes were blocked for 60 minutes in 5% non-fat skim milk (in phosphate-buffered saline containing 1% Tween 20 (PBST); 137mmol/L NaCl, 2.7mmol/L KCl, 10mmol/L Na_2HPO_4, 1.76mmol/L K_2HPO_4; pH 7.4) and sequentially incubated with either rabbit polyclonal anti-CFTR (1:1000 in 2% BSA/PBST; clone H182; *Santa Cruz Biotechnology* (Dallas, USA) cat# 10747) or mouse monoclonal anti-αtubulin (1:2000 in 2% BSA/PBST; clone DM1A; *New England Biolabs Canada* cat# 3873), followed by the appropriate peroxidase-labeled secondary antibody (1:20,000 in 2% BSA/PBST; *GE Healthcare Amersham* (Piscataway, USA) cat# NA934 and NA931). A standard chemiluminescence procedure was used to expose X-ray film; developed films were evaluated densitometrically using “Image J” software.

Table A. Patient characteristics: mesenteric artery samples.

Characteristic		Number
Age, years mean (range)		61 ± 3 (24 – 84)
Sex (Female/Male)		18/17
Surgical Indication	Cancer	27
	Crohn's disease	4
	Diverticular disease	4
	Fistula	3
	Ulcerative Colitis	2
	Multiple indications	3
Co- Morbidities	Hypertension	13
	Dyslipidemia	11
	Diabetes	2
	Obesity	4
	Deep Vein Thrombosis	2
	Myocardial Infarction	2
	Coronary Artery Disease	2
	Percutaneous Coronary Intervention	2
	Peripheral Vascular Disease	2
	Valvular Disease	1
	Arrhythmia	1
Drug Therapies	Angiotensin converting enzyme inhibitor	5
	Angiotensin 2 receptor antagonist	6
	Beta blocker	6
	Calcium-Channel Blocker	5
	Diuretic	7
	HMG-CoA reductase inhibitors	10
	Metformin	2
	Sulfonylureas	1
	Chemotherapy (prior to surgery)	3
Smoking status (non/present+former)		13/16

Table B. Patient characteristics: skeletal muscle artery samples.

Characteristic		Number
Age, years mean (range)		64 ± 1 (42 – 83)
Sex (Female/Male)		49/9
Surgical Indication	Coronary artery disease	58
	Mitral regurgitation	3
	Aortic Stenosis	3
	In-stent restenosis	8
	Cardiomyopathy	2
	Congestive heart failure	2
		44
Co-Morbidities	Hypertension	
	Dyslipidemia	56
	Myocardial infarction	16
	Percutaneous coronary intervention	12
	Stent	4
	Arrhythmia	1
	Aneurysm	4
	Peripheral Vascular Disease	9
	Diabetes	28
	Obesity	17
		39
Drug Therapies	Beta-blocker	
	Angiotensin converting enzyme inhibitor	29
	Angiotensin receptor antagonist	13
	Calcium-Channel Blocker	18
	Diuretic	13
	HMG-CoA reductase inhibitors	48
	Antiplatelet therapy	51
Smoking status (non/present+former)		18/40

Table C. Quantitative PCR primers targeting human mRNA transcripts.

Gene	Sequence (5′ to 3′)	Product Size (bp)	Efficiency	Accession no.
**Sphk1**	ATCTCCTTCACGCTGATGCT<br>CTCCAGACATGACCACCAGA	103	1.07	NM_021972.3<br>NM_182965.2<br>NM_001142601.1<br>NM_001142602.1
**SPP1**	TACTGCCTGTTCTGCTTCGG<br>ACATGACCAGCACCCAGATG	130	0.93	NM_030791
**$S1P_1R$**	AGCAGCAAGATGCGAAGC<br>CGATGAGTGATCCAGGCTTT	136	1.00	NM_001400
**$S1P_2R$**	GGAGTACCTGAACCCCAACA<br>GCAACAGAGGATGACGATGA	118	1.04	NM_004230
**$S1P_3R$**	GACTGCTCTACCATCCTGCC<br>GATGCGTGCGTAGAGGATCA	105	0.87	NM_005226
**CFTR**	AGCATTTGCTGATTGCACAG<br>ACTGCCGCACTTTGTTCTCT	101	1.03	NM_000492
**GAPDH**	CAATGACCCCTTCATTGACC<br>GACAAGCTTCCCGTTCTCAG	106	0.93	NM_002046
**G6PD**	GAGGCCGTGTACACCAAGAT<br>TCAGGGAGCTTCACGTTCTT	113	0.88	NM_000402.3
**HMBS**	TGCAACGGCGGAAGAAAA<br>AGCTGGCTCTTGCGGGTAC	66	1.08	BC019323.1

Acronyms: Sphk1 – sphingosine kinase 1; SPP1 – sphingosine-1-phosphate phosphatase 1; $S1P_1R$ – S1P receptor subtype 1; $S1P_2R$ – S1P receptor subtype 2; $S1P_3R$ – S1P receptor subtype 3; CFTR – cystic fibrosis transmembrane conductance regulator; GAPDH – glyceraldehyde 3-phosphate dehydrogenase; G6PD – glucose-6-phosphate dehydrogenase; HMBS – hydroxymethylbilane synthase.

Table D. Quantitative PCR primers targeting mouse mRNA transcripts.

Gene	Sequence (5′ to 3′)	Product Size (bp)	Efficiency	Accession no.
**Sphk1**	TGGGGCTATGACTTGGAAAG<br>CCAGGGAAGGTCCCTAAGAG	125	0.97	NM_011451.3<br>NM_025367.6<br>NM_001172473.1<br>NM_001172472.1<br>NM_001172475.1
**SPP1**	GAGCAACTTGCCGCTCTACTA<br>GGTCGAGATTCCAGATCCAGAA	101	0.93	NM_030750.3
**$S1P_1R$**	ATGGTGTCCACTAGCATCCC<br>CGATGTTCAACTTGCCTGTGTAG	112	0.93	NM_007901.5
**$S1P_2R$**	ATGGGCGGCTTATACTCAGAG<br>GCGCAGCACAAGATGATGAT	137	1.02	NM_010333.4
**$S1P_3R$**	ACTCTCCGGGAACATTACGAT<br>CAAGACGATGAAGCTACAGGTG	120	0.93	NM_010101.4
**CFTR**	CTGGACCACACCAATTTTGAGG<br>GCGTGGATAAGCTGGGGAT	162	1.05	NM_021050.2
**GAPDH**	AGGTCGGTGTGAACGGATTTG<br>TGTAGACCATGTAGTTGAGGTCA	123	0.94	NM_008084.2
**G6PD**	CACAGTGGACGACATCCGAAA<br>AGCTACATAGGAATTACGGGCAA	103	1.02	NM_008062
**HMBS**	CCCGTAACATTCCAAGAGGA<br>CCTGTGCCCTACAGACCAGT	147	1.08	NM_013551.2

Acronyms: Sphk1 – sphingosine kinase 1; SPP1 – sphingosine-1-phosphate phosphatase 1; $S1P_1R$ – S1P receptor subtype 1; $S1P_2R$ – S1P receptor subtype 2; $S1P_3R$ – S1P receptor subtype 3; CFTR – cystic fibrosis transmembrane conductance regulator; GAPDH – glyceraldehyde 3-phosphate dehydrogenase; G6PD – glucose-6-phosphate dehydrogenase; HMBS – hydroxymethylbilane synthase.

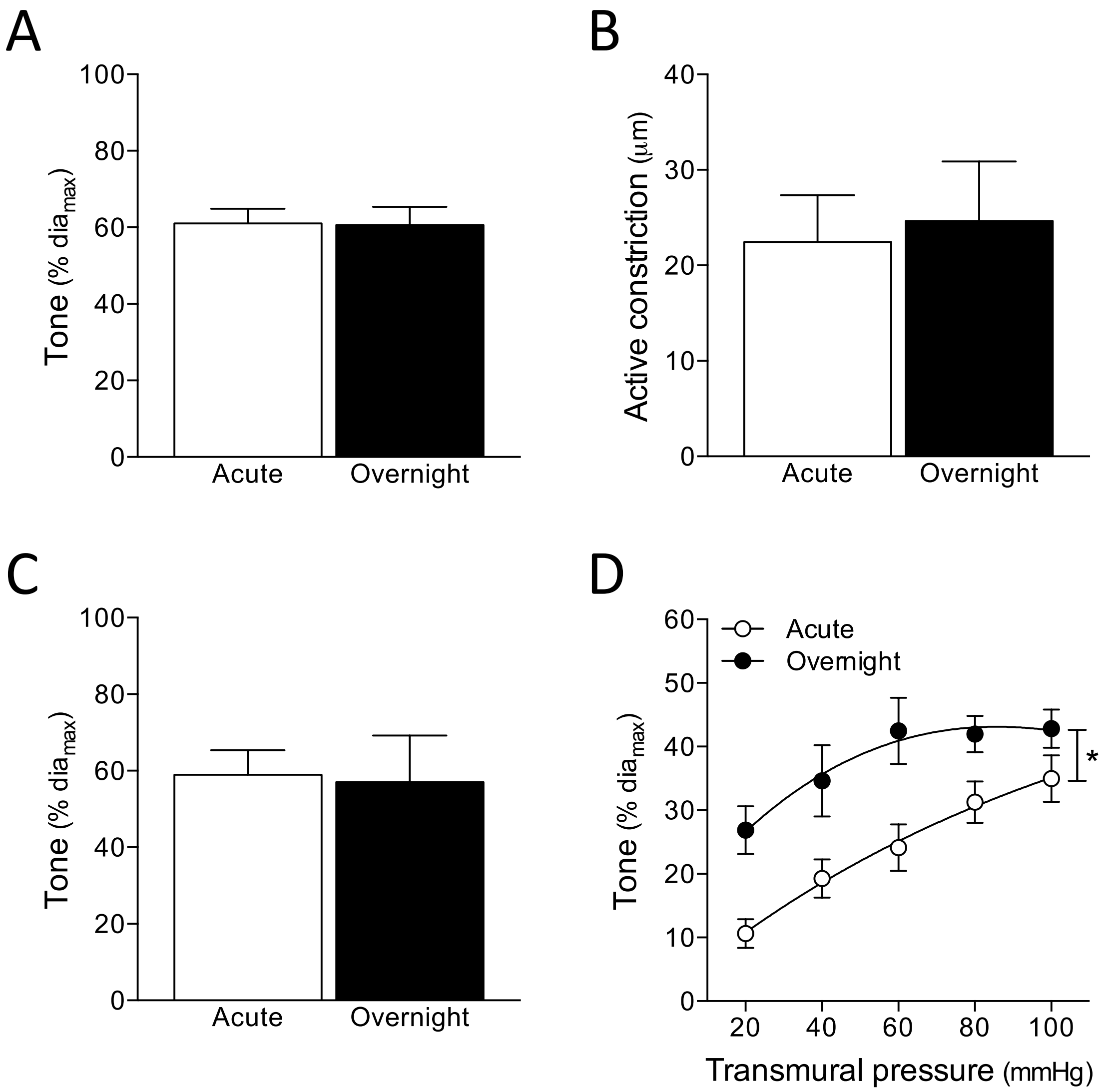

Fig A. Human resistance artery responses following overnight storage.

Initial experiments confirmed that storing isolated human mesenteric resistance arteries overnight (i.e., in MOPS buffer at 4°C) has no effect on (A) phenylephrine-stimulated vasoconstriction (1μmol/L) or (B) active myogenic constriction in response to a pressure step from 60mmHg to 100mmHg (Acute: dia_{max}=187±27μm, n=11 vessels from N=5 patients; Overnight: dia_{max}=219±22μm, n=9, N=7).

Storing skeletal muscle resistance arteries has no effect on (C) phenylephrine-stimulated vasoconstriction (Acute: dia_{max}=100±13μm, n=13, N=10; Overnight: dia_{max}=131±18μm, n=5, N=4); however, (D) storage augments myogenic tone (Acute: dia_{max}=105±13μm, n=13, N=9; Overnight: dia_{max}=131±18μm, n=5, N=3) over all pressures and flattens pressure-stimulated responses between 60-100mmHg (i.e., transmural pressure affects tone between 60-100mmHg in acutely-tested arteries, but not in stored arteries).

Dia_{max} is defined as the maximal diameter (under calcium free buffer conditions) at 60mmHg. Panels A, B and C were statistically compared with a Mann-Whitney test; Panel D was compared with a two-way ANOVA. * denotes $P<0.05$.

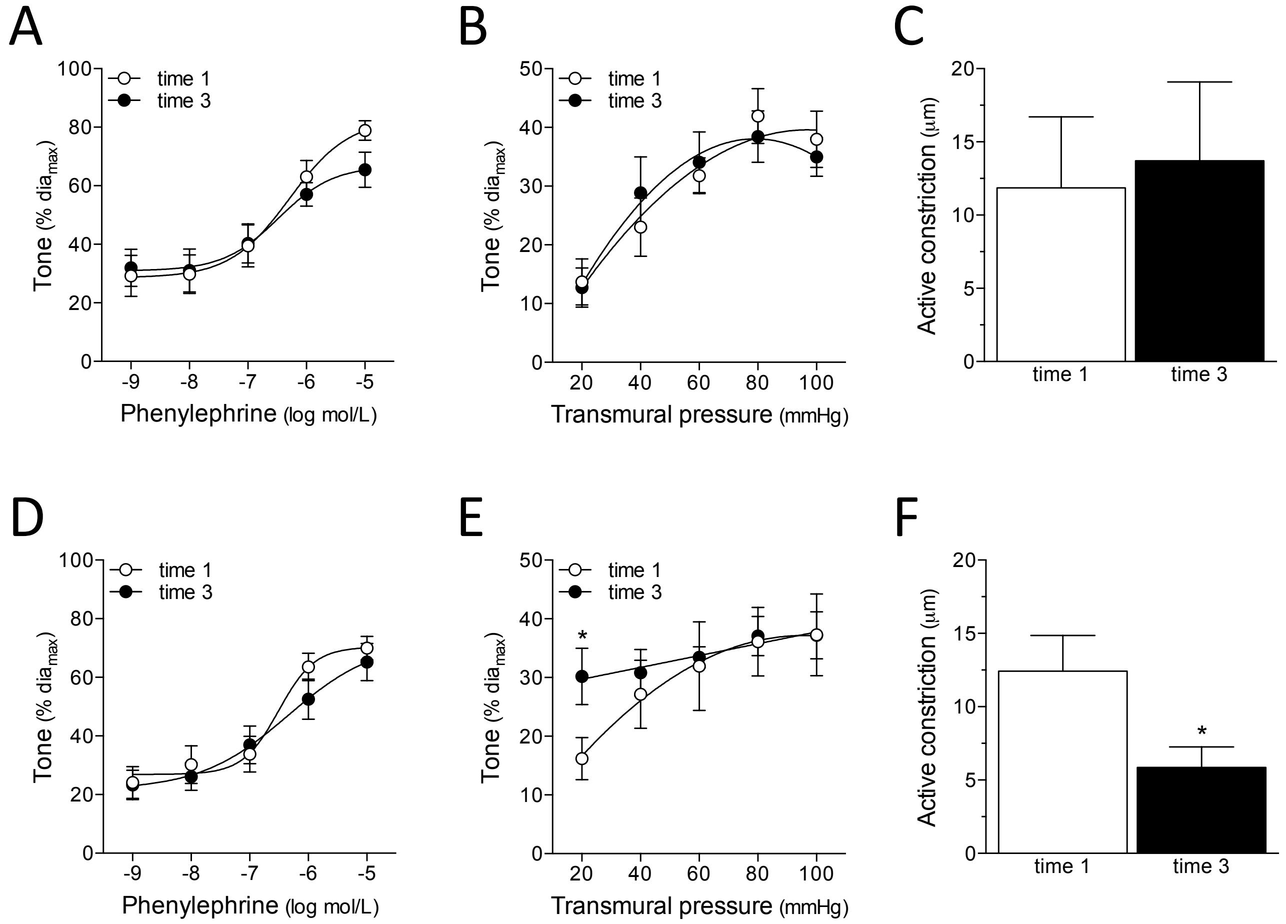

Fig B. Extended time controls in human mesenteric and skeletal muscle resistance arteries.

Functional parameters were initially assessed ("time 1"); the vessel was then incubated for 30 minutes under normal buffer conditions (i.e., the time period that corresponds to a typical inhibitor incubation requirement) and re-tested ("time 2"; data shown in Fig 1); this procedure was repeated, yielding functional assessments at "time 3". Note that responses for "time 1" are duplicated from Fig 1.

In human mesenteric resistance arteries, (A) PE-stimulated vasoconstriction (dia_{max}=173±33μm, n=7 vessels from N=5 patients), (B) myogenic tone (tone over a transmural pressure range of 20-100mmHg; dia_{max}=185±34μm, n=7, N=4) and (C) myogenic vasoconstriction (response to a pressure step from 60mmHg to 100mmHg; dia_{max}=105±15μm, n=7, N=3) are not different at "time 3", relative to the initial assessment at "time 1".

In human skeletal muscle resistance arteries, (D) PE-stimulated vasoconstriction (dia_{max}=104±13μm, n=10, N=10) remains comparable; however, both (E) myogenic tone (dia_{max}=122±21μm, n=9, N=8) and (D) myogenic vasoconstriction (dia_{max}=94±9μm,n=7, N=6) display differences at "time 3", relative to the initial assessment at "time 1".

Dia_{max} is defined as the maximal diameter (under calcium free buffer conditions) at 60mmHg. Myogenic tone (Panels A and D) and PE responses (Panels B and E) were statistically compared with a paired two-way ANOVA; active constriction measures (Panels C and F) were compared with a Wilcoxon test. * denotes P<0.05 relative to time 1 (paired comparisons).

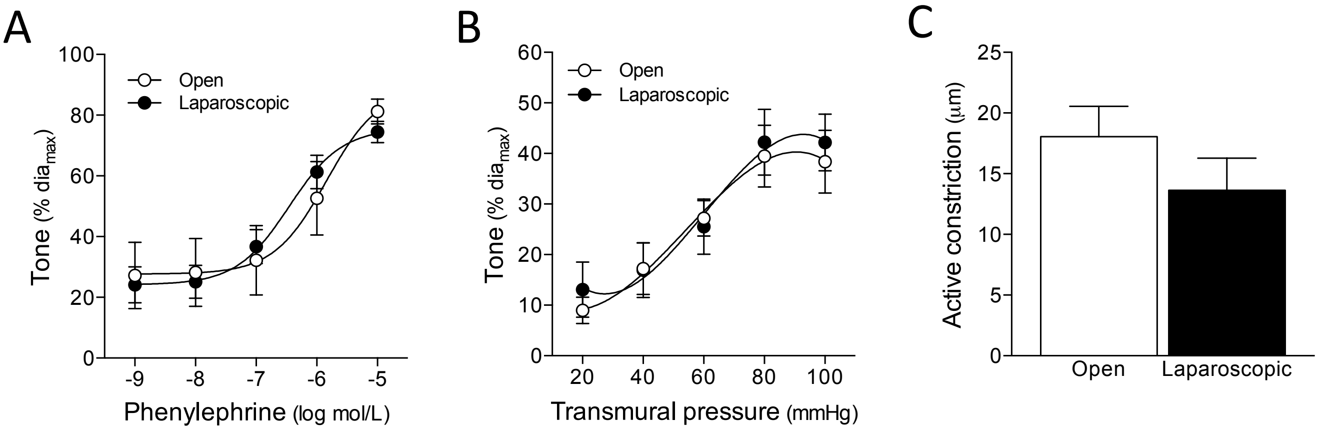

Fig C. Human mesenteric resistance artery responses are not influenced by the surgical procedure.

Open and laparoscopic surgical procedures yielded human mesenteric resistance arteries with similar (A) phenylephrine responses (Open: dia_{max}=148±43μm, n=6 vessels from N=5 patients; Laparoscopic: dia_{max}=130±18μm, n=10, N=6), (B) myogenic tone (Open: dia_{max}=135±24μm, n=6, N=5; Laparoscopic: dia_{max}=208±42μm, n=5, N=3) and (C) active myogenic vasoconstriction responses (Open: dia_{max}=178±14μm, n=36, N=16; Laparoscopic: dia_{max}=157±14μm, n=16, N=9).

Dia_{max} is defined as the maximal diameter (under calcium free buffer conditions) at 60mmHg. Myogenic tone (Panels A) and PE responses (Panels B) were statistically compared with a two-way ANOVA; active constriction measures (Panels C) were compared with a Mann-Whitney test.

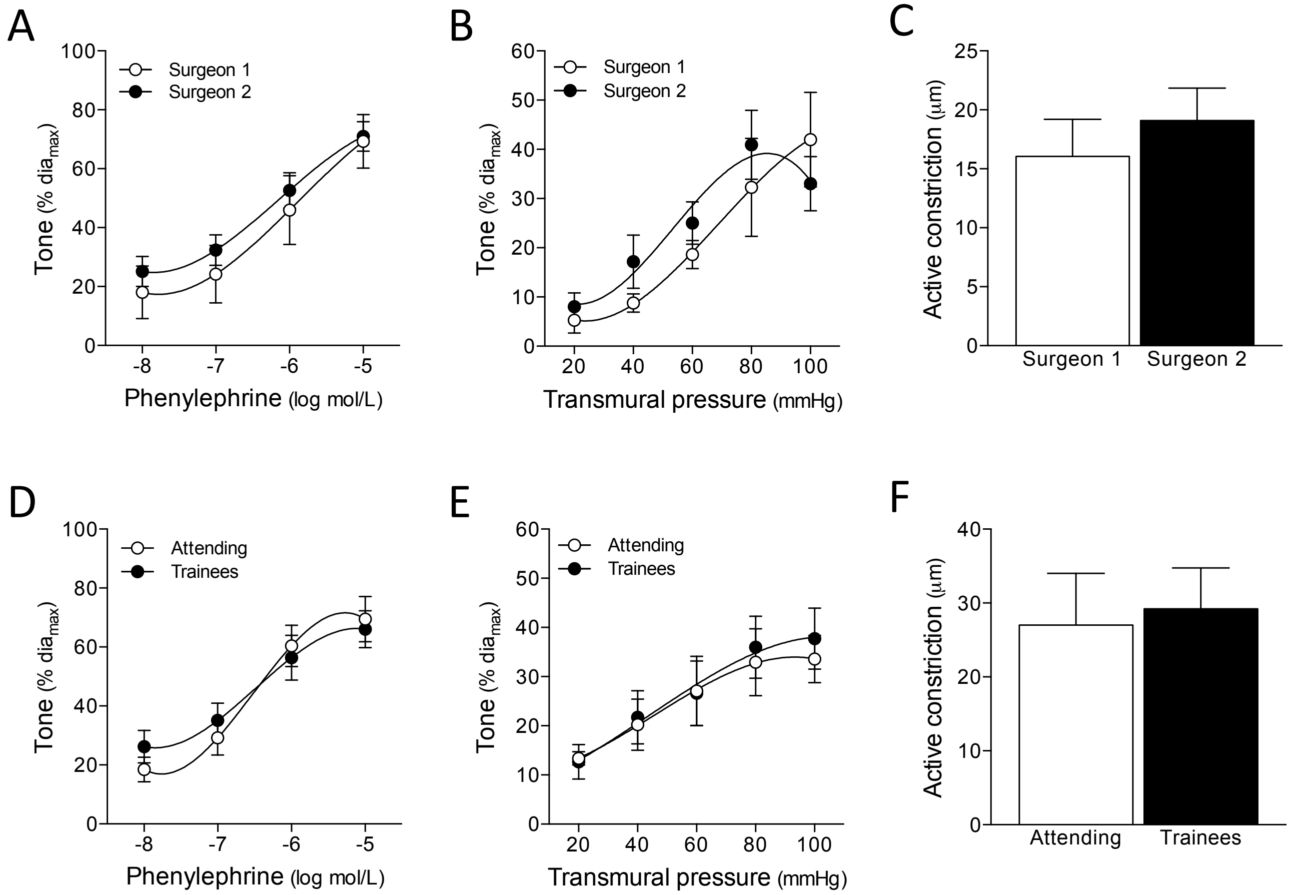

Fig D. Human resistance arteries have similar responses across attending surgeons.

Two collaborating surgeons provided the majority of mesenteric tissue samples. When grouped by the attending surgeon, human mesenteric resistance arteries displayed similar (A) phenylephrine responses (Surgeon 1: dia_{max}=134±26μm, n=8 vessels from N=7 patients; Surgeon 2: dia_{max}=164±24μm, n=11, N=7), (B) myogenic tone (Surgeon 1: dia_{max}=125±40μm, n=4, N=3; Surgeon 2: dia_{max}=167±36μm, n=6, N=5) and (C) active myogenic vasoconstriction (Surgeon 1: dia_{max}=184±20μm, n=23, N=10; Surgeon 2: dia_{max}=167±12μm, n=24, N=12).

Similarly, staff surgeons and their trainees (surgical fellows and medical residents) provided skeletal muscle samples. When grouped accordingly, isolated skeletal muscle resistance arteries displayed similar (D) phenylephrine responses (Attending: dia_{max}=130±21μm, n=6, N=4; Trainee: dia_{max}=112±16μm, n=9, N=7), (E) myogenic tone (Attending: dia_{max}=130±21μm, n=6, n=5; Trainee: dia_{max}=108±15μm, n=10, N=8) and (F) active myogenic vasoconstriction (Attending: dia_{max}=122±24μm, n=5, N=4; Trainee: dia_{max}=109±17μm, n=9, N=8).

Dia_{max} is defined as the maximal diameter (under calcium free buffer conditions) at 60mmHg. Myogenic tone (Panels A and D) and PE responses (Panels B and E) were statistically compared with a two-way ANOVA; active constriction measures (Panels C and F) were compared with a Mann-Whitney test.

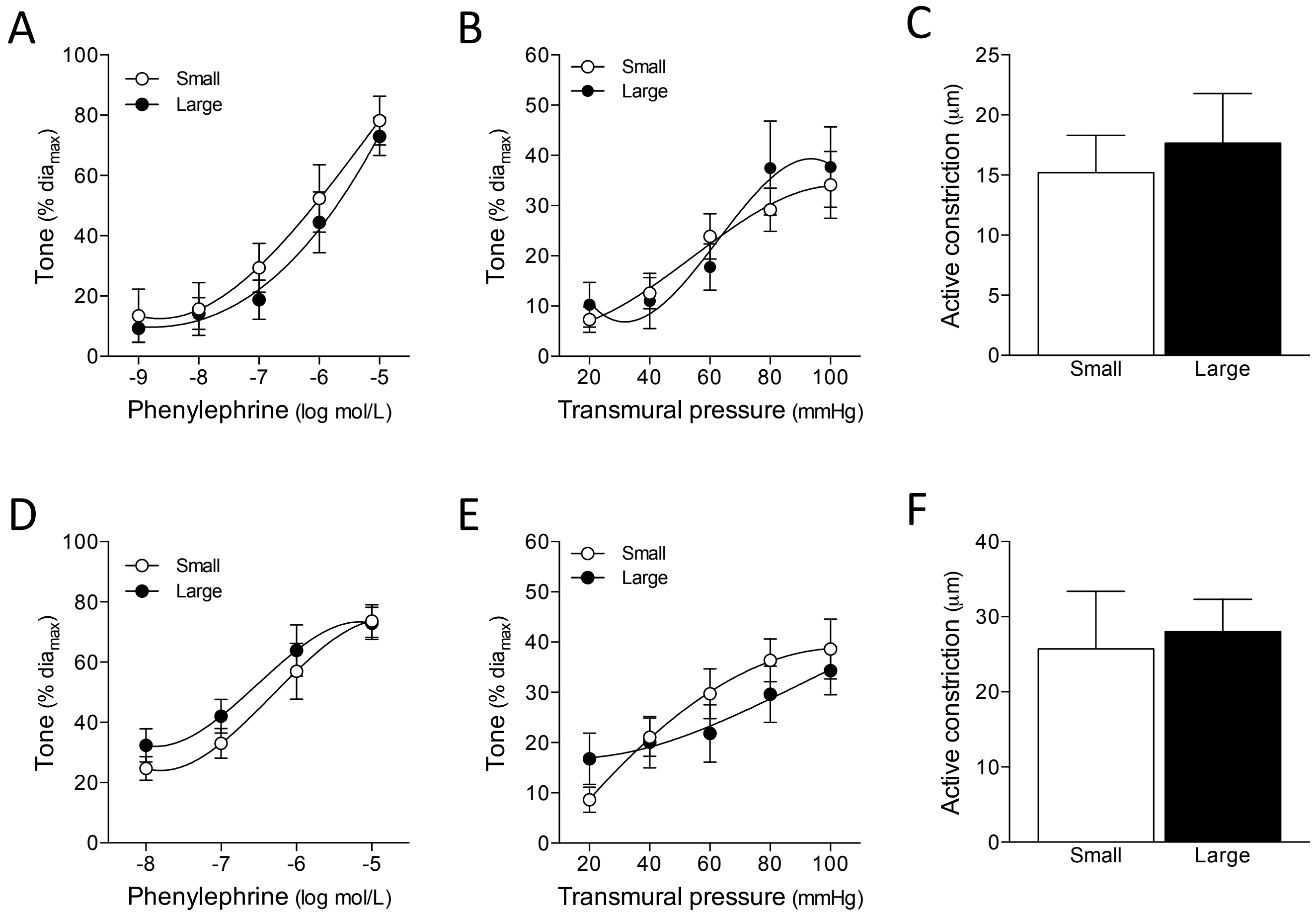

Fig E. Vessel diameter does not influence resistance artery responsiveness.

Human mesenteric and skeletal muscle resistance arteries were segregated into the top and bottom quartiles by size (for all panels, Small dia_{max} and Large dia_{max} are statistically different). Small and large human mesenteric resistance arteries possessed similar (A) phenylephrine responses (Small dia_{max}=81±6µm, n=5 vessels from N=4 patients; Large dia_{max}=266±17µm, n=5, N=5) (B) myogenic tone (Small dia_{max}=112±11µm, n=6, N=6; Large dia_{max}=249±16µm, n=6, N=5) and (C) active myogenic vasoconstriction (Small dia_{max}=87±5µm, n=15, N=11; Large dia_{max}=269±12µm, n=15, N=10).

Likewise, small and large human skeletal resistance arteries possessed similar (D) phenylephrine responses (Small Dia_{max} 67.3±6.1µm, n=7, N=4; Large dia_{max}=133.1±16.5µm, n=7, N=6), (E) myogenic tone (Small Dia_{max}=67.3±6.1µm, n=7, N=4; Large dia_{max}=133.1±16.5µm, n=7, N=6) and (F) active myogenic vasoconstriction (Small Dia_{max}=67.3±6.1µm, n=7, N=4; Large dia_{max} 144±21.7µm, n=5, N=5).

Dia_{max} is defined as the maximal diameter (under calcium free buffer conditions) at 60mmHg. Myogenic tone (Panels A and D) and PE responses (Panels B and E) were statistically compared with a two-way ANOVA; active constriction measures (Panels C and F) were compared with a Mann-Whitney test.

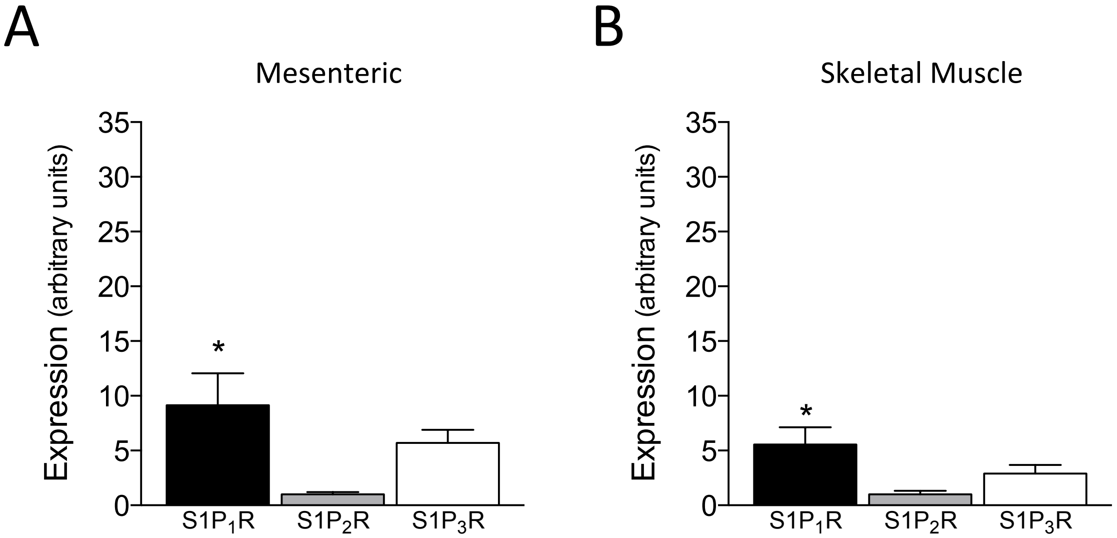

Fig F. Mouse resistance artery sphingosine-1-phosphate receptor mRNA expression.

Using quantitative PCR, the relative mRNA expression of S1P receptor subtypes 1-3 ($S1P_1R$, $S1P_2R$ and $S1P_3R$) was determined in mouse (A) mesenteric (n=6) and (B) cremaster skeletal muscle (n=7) resistance arteries. $S1P_1R$ expression is statistically higher than $S1P_2R$ in both artery types. * denotes $P<0.05$ relative to $S1P_2R$ (Friedman ANOVA).

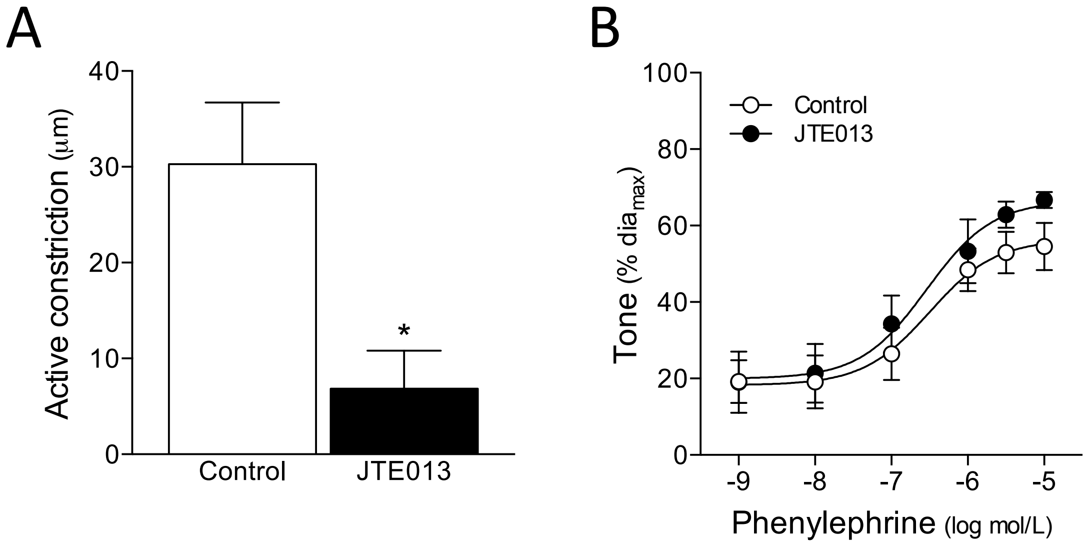

Fig G. JTE013 attenuates myogenic vasoconstriction but not phenylephrine-stimulated vasoconstriction.

In human mesenteric arteries (dia_{max}=148±30µm, n=7 vessels from N=4 patients), S1P receptor antagonism (10nmol/L JTE013; 30 minutes) (A) attenuates active myogenic vasoconstriction, but (B) has no significant effect on phenylephrine (PE)-stimulated vasoconstriction (dia_{max}=140±34µm, n=6, N=4). * denotes P<0.05 for paired data comparisons.

Dia_{max} is defined as the maximal diameter (under calcium free buffer conditions) at 60mmHg. Panel A is statistically compared with a Wilcoxon test; Panel B is compared with a paired two-way ANOVA. * denotes P<0.05.

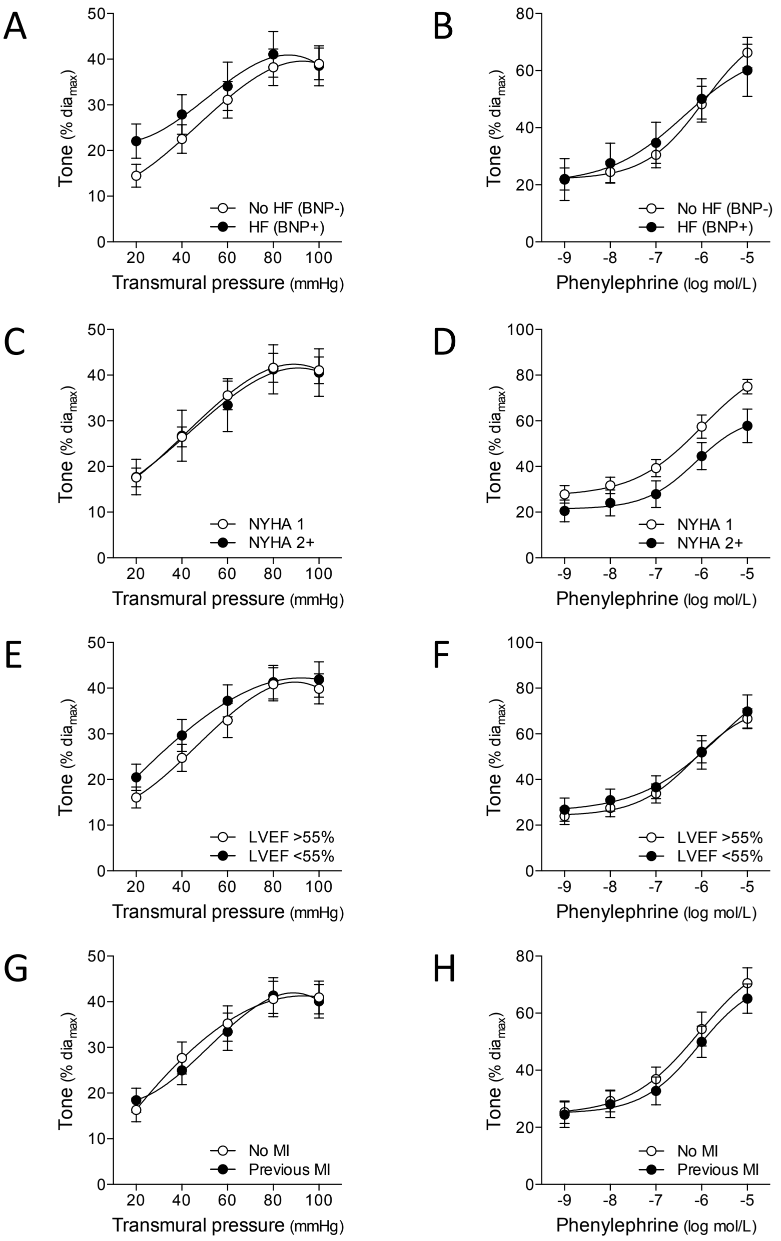

Fig H. Human skeletal muscle resistance artery responses segregated by heart failure severity.

Human skeletal muscle resistance artery myogenic tone (left panels) and phenylephrine-stimulated vasoconstriction (right panels) do not differ when segregated by (A,B) a positive result for heart failure (HF), based on "N-terminal of prohormone B-type natriuretic peptide (BNP)" serum levels (No HF: dia_{max}=131±14µm, n=15 vessels from N=9 patients; HF: dia_{max}=104±10µm, n=8, N=7); (C,D) New York Heart Association (NYHA) Functional Class 1 versus Functional Classes 2 and above (NYHA 1: dia_{max}=119±10µm, n=20, N=14; NYHA 2+: dia_{max}=120±18µm, n=11, N=8); (E,F) reduced left ventricular ejection fraction (LVEF), with normal defined as 55% (LVEF>55%: dia_{max}=122±10µm, n=22, N=15; LVEF<55%: dia_{max}=116±17µm, n=10, N=8); or (G,H) previous documentation of myocardial infarction (MI) in the patient record (No MI: dia_{max}=106±10µm, n=15, N=11; Previous MI: dia_{max}=133±13µm, n=17, N=12). Dia_{max} is defined as the maximal diameter (under calcium free buffer conditions) at 60mmHg. All panels utilize a two-way ANOVA for statistical comparisons.

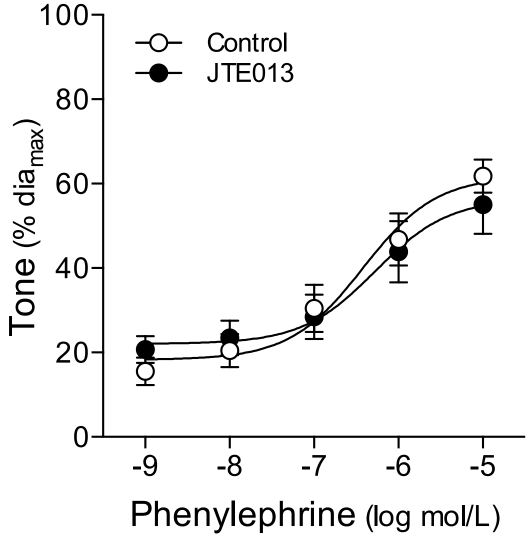

Fig I. JTE013 does not attenuate phenylephrine-stimulated vasoconstriction in human skeletal muscle resistance arteries.

At a concentration of 0.1nmol/L, JTE013 (30 minute treatment) does not affect phenylephrine-stimulated vasoconstriction in human skeletal muscle resistance arteries (dia_{max}=145±23µm n=9 vessels from N=7 patients). Dia_{max} is defined as the maximal diameter (under calcium free buffer conditions) at 60mmHg. Data are statistically compared with a paired two-way ANOVA.

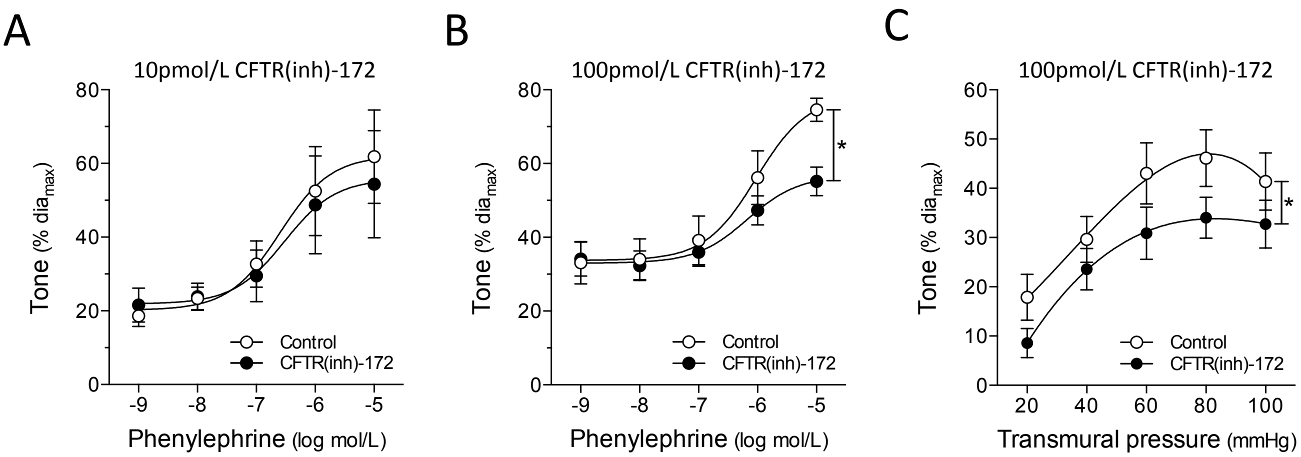

Fig J. CFTR inhibition dose-dependently attenuates phenylephrine-stimulated vasoconstriction and myogenic responses in human skeletal muscle resistance arteries.

(A) At a concentration of 10pmol/L, $CFTR_{(inh)}$-172 (30 minute treatment) does not affect phenylephrine-stimulated vasoconstriction in human skeletal muscle resistance arteries (dia_{max}=109±20μm, n=5 vessels from N=3 patients). However, at 100pmol/L, $CFTR_{(inh)}$-172 (30 minute treatment) attenuates both (B) phenylephrine-stimulated vasoconstriction (dia_{max}=122±21μm, n=9, N=5) and (C) myogenic responsiveness (dia_{max}=105±22μm, n=7, N=5).

Dia_{max} is defined as the maximal diameter (under calcium free buffer conditions) at 60mmHg. All data are statistically compared with a paired two-way ANOVA. * denotes P<0.05.

Supplement References

1 Meissner A, Yang J, Kroetsch JT, Sauvé M, Dax H, Momen A, et al. Tumor Necrosis Factor-α-Mediated Downregulation of the Cystic Fibrosis Transmembrane Conductance Regulator Drives Pathological Sphingosine-1-Phosphate Signaling in a Mouse Model of Heart Failure. Circulation. 2012;125: 2739-2750.
